# Expression Analysis of *Gnrh1* and *Gnrhr1* in Spermatogenic Cells of Rat

**DOI:** 10.1155/2015/982726

**Published:** 2015-03-12

**Authors:** Vincenza Ciaramella, Rosanna Chianese, Paolo Pariante, Silvia Fasano, Riccardo Pierantoni, Rosaria Meccariello

**Affiliations:** ^1^Dipartimento di Medicina Sperimentale Sezione “F. Bottazzi”, Seconda Università di Napoli, Via Costantinopoli 16, 80138 Napoli, Italy; ^2^Dipartimento di Scienze Motorie e del Benessere, Università di Napoli Parthenope, Via Medina 40, 80133 Napoli, Italy

## Abstract

Hypothalamic Gonadotropin Releasing Hormone (GnRH), *via* GnRH receptor (GnRHR), is the main actor in the control of reproduction, in that it induces the biosynthesis and the release of pituitary gonadotropins, which in turn promote steroidogenesis and gametogenesis in both sexes. Extrabrain functions of GnRH have been extensively described in the past decades and, in males, local GnRH activity promotes the progression of spermatogenesis and sperm functions at several levels. The canonical localization of *Gnrh1* and *Gnrhr1* mRNA is Sertoli and Leydig cells, respectively, but ligand and receptor are also expressed in germ cells. Here, we analysed the expression rate of *Gnrh1* and *Gnrhr1* in rat testis (180 days old) by quantitative real-time PCR (qPCR) and by *in situ* hybridization we localized *Gnrh1* and *Gnrhr1* mRNA in different spermatogenic cells of adult animals. Our data confirm the testicular expression of *Gnrh1* and of *Gnrhr1* in somatic cells and provide evidence that their expression in the germinal compartment is restricted to haploid cells. In addition, not only Sertoli cells connected to spermatids in the last steps of maturation but also Leydig and peritubular myoid cells express *Gnrh1*.

## 1. Introduction

One of the most intriguing matters in reproductive endocrinology is that, in both mammalian and nonmammalian vertebrates, molecules originally identified in the brain as neuropeptides/neurohormones exert their activity in extrabrain tissues, in particular in the gonads, which express the corresponding receptors [1, 2]. Notable examples are Neuropeptide Y, Corticotropin Releasing Factor, Gonadotropin Inhibiting Factor, and Kisspeptin [1, 3, 4]. The activity of Gonadotropin Releasing Hormone (GnRH),* via* GnRH receptor (GnRHR), was the first to be reported in ovary and testis from fish to mammals [2, 5] as from the end of 1970s.

GnRH is the gatekeeper of reproductive functions in both sexes. This decapeptide is synthesized in the hypothalamus and is released in the median eminence to trigger the discharge of pituitary gonadotropins (Follicle Stimulating Hormone and Luteinizing Hormone (FSH and LH, resp.)). Through the main circulation, FSH and LH reach the gonads and sustain gametogenesis and steroid biosynthesis [5]. GnRH was originally isolated in the ‘70s from the hypothalamus of pig and sheep [6, 7]. Nowadays, GnRH saga comprises at least 25 GnRH molecular forms and three GnRHRs, seven-transmembrane G-coupled receptors that exhibit a wide range of subtypes [5, 8–11]. In mammals, including human, two GnRH molecular forms (GnRH1 and GnRH2) and two GnRHRs (GnRHR1 and GnRHR2) have been characterized [5, 11]. Whether* GNRHR2* in humans is a functional or a remnant gene is still an unresolved matter of debate [9]. However, the main role of GnRH1 is the communication between basal hypothalamus and pituitary gland. By contrast, GnRH2, besides a supposed hypophysiotropic activity, is mainly produced in the hindbrain and exerts a neurotransmitter and/or neuromodulatory role in the control of food intake, energy balance, sexual behavior, and stress in response to many environmental cues. A third GnRH molecular form exhibiting neuromodulator activity has been only detected in the telencephalon of teleost fish. Three different GnRHRs have been detected in fish and in amphibians [5, 8, 11].

Spermatogenesis is a complex process in which mitosis, meiosis, and differentiation coexist in order to produce high quality sperm under the control of hormonal milieu. Data in mammalian and nonmammalian animal models and in cell lines have provided evidence that steroid biosynthesis and gonocyte as well as spermatogonia proliferation, spermatogenesis progression, germ cell apoptosis, sperm release, and fertilization all require the local activity of GnRH (for review see [2, 5, 12–14]). Thus, in the testis, GnRH and GnRHR are deeply involved in the autocrine and paracrine routes that modulate the communications between somatic and germ cells [2], acting in current with several local biomodulators [5, 15–21].

Foetal expression of GnRH1 and GnRHR1 has been reported during the ontogenesis of rat gonads [22] and in mouse testis high GnRH1 activity has been observed from pubertal stages to the adult, with a decline during senescence [3]. Leydig cells are the main target of GnRH activity [12, 23, 24]; GnRH1 has been detected in the interstitial fluid of rat testis [25] and competitive binding studies, immunohistochemistry, and* in situ* hybridization have suggested that Sertoli cells may represent the main source of GnRH (for review see [5, 12, 26]). Besides the canonical localization in somatic cells, in rat and human* Gnrh1/GNRH1* and* Gnrhr1/GNRHR1* transcripts have also been localized in germ cells [27]. In human, a* GNRHR2* transcript is expressed in haploid postmeiotic cells and in mature sperm as well [28]. Consistently, in mouse and rat, Northern blot analysis has revealed the presence of several forms of* Gnrhr1* mRNA in isolated germ cells [29]. In particular, in rat, spermatogenic cells of some seminiferous tubules also express* Gnrh1* and* Gnrhr1* [27]. However, at present, little is known about the localization of GnRH1 and its receptor in different spermatogenic cells. Here, we fill this gap by reporting the localization of* Gnrh1* and* Gnrhr1* mRNA during spermatogenesis, in adult rats, focusing on the localization of* Gnrh1* and* Gnrhr1* transcripts during the steps of the spermiogenesis.

## 2. Materials and Methods

### 2.1. Animals and Tissue Collection

Wistar rats (*Rattus norvegicus*) were housed under definite conditions (12D : 12L) and given free access to standard food and water* ad libitum*. Animals at 180 days* postpartum* (dpp) were sacrificed by decapitation under ketamine anesthesia (100 mg/kg i.p.) in accordance with local and national guidelines covering experimental animals. For each animal testes were dissected; one testis was fixed in Bouin's fluid and processed for* in situ* hybridization; one was quickly frozen by immersion in liquid nitrogen and stored at −80°C until RNA extraction. Additionally, adult rat brain was also dissected, frozen, and stored at −80°C to be used as a positive control in qPCR analysis. This project was approved by the Italian Ministry of Education, University and Research (MIUR). Procedures involving animal care were conducted in accordance with the Guide for Care and Use of Laboratory Animals (National Institutes of Health guide).

### 2.2. Total RNA Extraction and cDNA Preparation

Total RNA was extracted from* R. norvegicus* tissues (testis and brain) using Trizol Reagent (1 mL/50–100 mg tissue) (Invitrogen Life Technologies, Paisley, UK) according to the manufacturer's instructions. Total RNA was treated for 30 min at 37°C with Dnase I (10 U/sample) (Amersham Pharmacia Biotech) to eliminate any contamination of genomic DNA. RNA purity and integrity were determined by spectrophotometer analyses at 260/280 nm and by electrophoresis. Complementary DNA (cDNA) was obtained by reverse transcription using 5 *μ*g of total RNA, 0.5 *μ*g of oligo dT_(18)_, 0.5 mM dNTP mix, 5 mM DTT, 1x first-strand buffer (Invitrogen Life Technologies), 40 U RNase Out (Invitrogen Life Technologies), and 200 U SuperScript-III RnaseH^−^ Reverse Transcriptase (Invitrogen Life Technologies) in a final volume of 20 *μ*L, following the manufacturer's instructions. As negative control, total RNA not treated with reverse transcriptase was used.

### 2.3. Cloning of* Gnrh1* and* Gnrhr1*

To clone* Gnrh1* and* Gnrhr1*, 1 *μ*L of diluted (1 : 5) cDNA was used for standard PCR analysis in combination with 10 pmol of oligonucleotide primers designed on* R. norvegicus* nucleotide sequence (*Gnrh1*: 5′-agcactggtcctatgggttg-3′ and 5′-gggccagtgcatctacatct-3′;* Gnrhr1*: 5′-cagctttcatgatggtggtg-3′ and 5′-ttcagctgtagtttgcgtgg-3′). The predicted amplificate sizes were 221 and 370 bp, for* Gnrh1* and* Gnrhr1*, respectively. PCR conditions were 94°C, 5 min, 1 cycle; 94°C, 30 s, 58°C, 30 s, 72°C, 45 s, 40 cycles; 72°C, 7 min. PCR products were subcloned in pGEM-T Easy Vector (Promega Corp., Madison, WI). DH5*α* high-efficiency competent cells were transformed and recombinant colonies were identified by blue/white colour screening. Recombinant plasmid DNA was extracted by using the QIAprep Spin Miniprep kit (Qiagen, Valencia, CA), and the insert sizes were controlled by restriction analysis with EcoRI (Fermentas, St. Leon-Rot, Germany) and then they were sequenced on both strands by Primm Sequence Service (Primm Srl, Naples Italy).

### 2.4. Riboprobes Synthesis and* In Situ* Hybridization

Specific riboprobes were synthesized by* in vitro* transcription using cDNA fragments of rat* Gnrh1* and* Gnrhr1*, 221 and 370 bp, respectively, cloned previously in pGEM-T Easy Vector (Promega Corp.). The sense (control) and antisense complementary RNA (cRNA) probes were transcribed with T7 and SP6 RNA polymerases on plasmids and linearized with the appropriate restriction enzymes (*Sal*I or* Nco*I) using DIG-uridine triphosphate (UTP) RNA labeling mix (Roche Diagnostics, Mannheim, Germany) as recommended by the manufacturer.

For histological observation, rat testes were fixed in Bouin's fluid, dehydrated in ethanol, cleared in xylene, and embedded in paraffin. Tissue sections (5 *μ*m) were stained with hematoxylin-eosin to assess sample quality.* In situ* hybridization was performed as follows. In brief, sections were subjected to the treatment with 10 *μ*g/mL proteinase K (Sigma Aldrich) in 20 mM Tris-HCl; then hybridization was performed overnight at 60°C in a humidified chamber using 100 *μ*L hybridization buffer (40% deionized formamide, 5x SSC, 1x Denhardt's solution, 100 *μ*g/mL sonicated salmon sperm DNA, 100 *μ*g/mL tRNA, and 100 ng digoxigenin-labeled cRNA probe). Finally, sections were incubated for 30 min at 37°C in RNase buffer (0.5 M NaCl, 10 mM Tris-HCl, pH 7, and 5 mM EDTA) containing 20 *μ*g/mL RNase A. Slides were observed under a light microscope (Leica LEITS DMRB; Leica Microsystem, Milan, Italy) and images were captured using a high-resolution digital camera (Leica MC 170 HD, Software Leica Application Suite 4.3).

### 2.5. Quantitative Real-Time PCR (qPCR)

Quantitative mRNA analysis was conducted on testis and brain to evaluate* Gnrh1* and* Gnrhr1* expression. All qPCRs were prepared in a final volume of 20 *μ*L containing 1 *μ*L of 1 : 5 diluted cDNA, 0.5 *μ*M of each primer, and 10 *μ*L of SSo Fast EvaGreen supermix (Bio-Rad). Assays were run twice in duplicate using the Mastercycler CFX-96 (Bio-Rad); a negative control in which cDNA was replaced by water was also included. All assays included a melting curve analysis for which all samples displayed single peaks for each primer pair. Genes of interest were normalized to the reference gene *β-actin* [5′-agatgacccagatcatgtttgaga-3′ and 5′-accagaggcatacagggacaa-3′; *T* (°C) annealing 56°C; amplificate size 86 bp]; the relative quantification of the mRNA levels was performed using the comparative Cq method with the formula 2^−ΔΔCq^. Data were then reported as mean fold change ± SD over the minimal value arbitrarily assigned to a reference sample (brain sample). ANOVA followed by Duncan's test for multigroup comparison was carried out to assess the significance of differences.

## 3. Results

### 3.1. Expression of* Gnrh1* and* Gnrhr1* Transcripts in* R. norvegicus*

**Figure 1:**
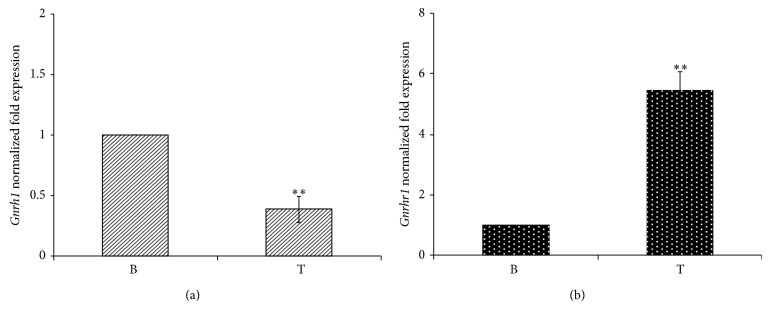
*Gnrh1* (a) and* Gnrhr1* (b) expression by qPCR in* R. norvegicus* tissues. B, brain; T, testis. Data are reported as mean fold change ± SD over the value one arbitrarily assigned to brain sample. Asterisks indicate statistically significant differences (*P* < 0.01), and data are representative of three separate experiments at least (*n* = 6).

Standard RT-PCR was preliminarily carried out on adult rats in order to clone* Gnrh1* and* Gnrhr1* cDNA fragments. Bands of the predicted size (221 and 370 bp for* Gnrh1* and* Gnrhr1*, resp.) were observed in the testis. Therefore, the abundance of* Gnrh1* and* Gnrhr1* was evaluated by qPCR analysis on total RNA from adult male gonads and brain of* R. norvegicus* (Figures 1(a) and 1(b)). The analysis revealed the presence of the transcripts,* Gnrh1* and* Gnrhr1*, in the testis as well as in the brain (positive control). In particular, the expression of* Gnrh1* in the testis was significantly lower than in the brain (*P* < 0.01) (Figure 1(a)) and that of* Gnrhr1* increased 5.46-fold in the testis compared to the brain (*P* < 0.01) (Figure 1(b)).

### 3.2. Localization of* Gnrh1* and* Gnrhr1* in* R. norvegicus* Testis

**Figure 2:**
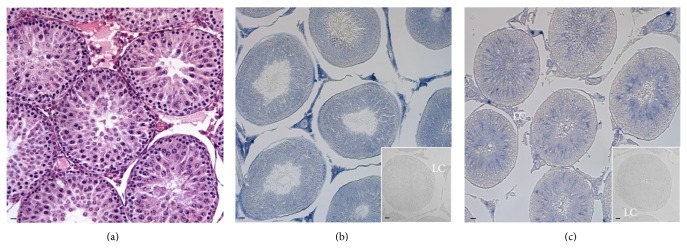
Section of adult* R. norvegicus* testis analyzed by hematoxylin-eosin staining (a) as well as by* in situ* hybridization for* Gnrh1* (b) and* Gnrhr1* (c) treated with antisense ((b), (c)) and sense ((b), (c) insets) probes. Localization of* Gnrh1* and* Gnrhr1* was observed in the interstitial Leydig cells (LC) and inside the seminiferous tubule. Scale bars: 20 *μ*m. The results are representative of one of three assays.

*Gnrh1* and* Gnrhr1* transcripts were localized by* in situ* hybridization in rat testis at 180 dpp (Figure 2) during the spermatogenesis. The stages were classified according to Leblond and Clermont 1952 [30].

**Figure 3:**
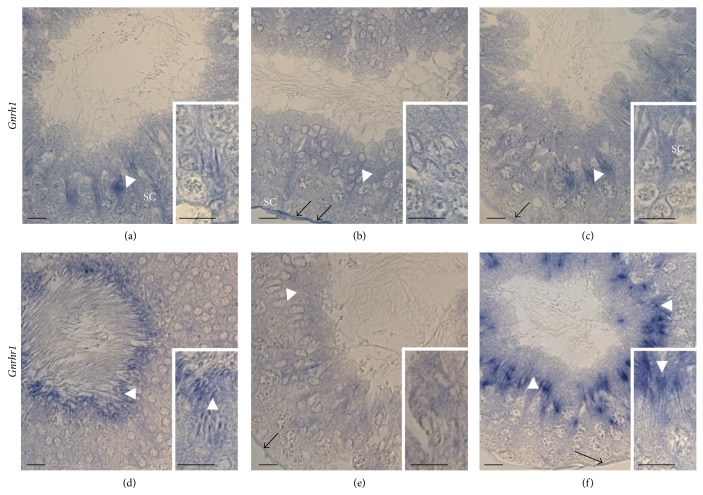
Localization of* Gnrh1* and* Gnrhr1* by* in situ* hybridization in rat testis during the stage of the spermatogenetic cycle ((a)–(f)). The blue staining indicates the positive cells; few of them, representative of the different cell types, are pointed out by white arrowhead, SPT ((a)–(f)); black arrow, peritubular myoid cells ((b), (c), (e), and (f)); SC, Sertoli cells. The insets are shown in 100x magnification. Scale bars: 20 *μ*m. The results are representative of one of three assays.

*Gnrh1* mRNA was strongly localized in the interstitial Leydig cells (LC) (Figure 2(b)) and in spermatids (SPT) in acrosome phase, Stages IX–XII (Figure 3(b)); the signal was also detected in Sertoli cells (SC) connected with SPT during the maturation phase, Stages V–VII (steps 17–19) (Figures 3(b) and 3(c)). Besides,* Gnrh1* mRNA outlined each seminiferous tubule, marking peritubular myoid cells (Figures 3(b) and 3(c)).

*Gnrhr1* mRNA was mainly localized in SPT from Stages III to V (steps 16-17) (Figure 3(d)), when SPT are in maturation phase, until Stages XII-XIII, when SPT are in the acrosome phase (Figures 3(e) and 3(f)); a weaker signal was also observed in the interstitial compartment (LC) (Figure 2(c)).

Quiescent primary SPG (ISPG) and pachytene or leptotene spermatocytes (SPC) did not reveal any positivity. No labeling for* Gnrh1* or* Gnrhr1* was detected in slides incubated with sense cRNA probes (*insets in* Figures 2(b) and 2(c)).

**Figure 4:**
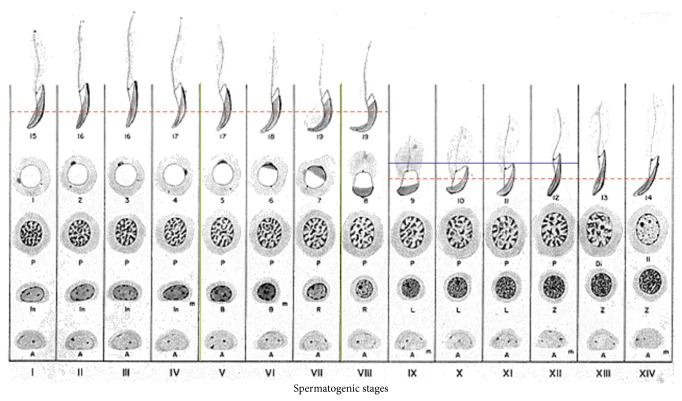
Schematic representation of* Gnrh1* and* Gnrhr1* expression in the germinal epithelium during a complete spermatogenetic cycle (modified from [45]). Columns show the sequence of germ cell generations observed in rat seminiferous tubule at that stage (I–XIV Stages). Spermatogonia: A (type A), In (intermediate), B (type B), R (resting, final DNA replication), and m (mitosis of spermatogonia). Primary spermatocytes: L (leptotene), Z (zygotene), P (pachytene), Di (diakinesis), and II (secondary spermatocytes). Phases 1–19 indicate spermatids differentiation (spermiogenesis). Horizontal lines indicate localization window of* Gnrh1* and* Gnrhr1,* blue line and red dotted line, respectively. Vertical green lines indicate the expression window of* Gnrh1* in Sertoli cells.

A schematic representation of* Gnrh1* and* Gnrhr1* expression in the germinal epithelium during the spermatogenetic cycle is represented in Figure 4.

## 4. Discussion

Local activity of GnRH has been reported in male and female gonads in several animal models from molluscs to vertebrates, human included [2, 5, 13, 14, 26]. In this respect, the use of GnRH agonists and antagonists in cell lines,* in vivo* and* in vitro,* has provided evidences of GnRH involvement in the control of Leydig cells ontogenesis and activity, spermatogenesis progression, sperm release, and function ([2, 5, 13, 14, 26] and references therein).

Present data confirm earlier findings that both* Gnrh1* and* Gnrhr1* are expressed in rodent testis as well as in brain used as positive control. In our study total RNA was extracted from whole brain and testis and the same amount of cDNA was used for cDNA preparation. Since it is well known that in the brain* Gnrh1* and* Gnrhr1* are expressed in discrete brain areas and not all over the tissue [5], a dilution effect on the expression of* Gnrh1* and* Gnrhr1* might be postulated in the brain. Despite that, present data confirm that the brain is the major source of GnRH1. By contrast, the expression levels of* Gnrhr1* here observed are higher in the testis than in the whole brain. Consistently previous results obtained by Northern blot, a less sensitive technique than qPCR, revealed that in rodents* Gnrhr1* is highly expressed in the pituitary gland, the tissue mainly responsive to GnRH1, but also in whole testis and germ cells [29, 31].

By* in situ* hybridization we have localized* Gnrh1* and* Gnrhr1* inside the testis throughout the spermatogenic cycle, which in rat comprises XIV different cellular associations, with newly formed and older spermatids overlapping during the first eight stages of development [30].

In general the communications between Leydig and Sertoli cells and between Sertoli and germ cells ensure an optimal environment for the progression of germ cells and are fundamental tools to gain a successful spermatogenesis [12]. Hypogonadal (*hpg*) mice, a natural model with arrested reproductive development due to a congenital deficiency in GnRH synthesis leading to markedly reduced production of gonadotropins, are infertile [32]. The administration of androgens is sufficient to induce spermatogenesis in* hpg* mice, but the administration of recombinant human FSH, whose main targets in testis are Sertoli cells, induces the proliferation of spermatogonia and spermatogenesis progression until spermatocytes [33, 34]. Similarly, the lack of LH signalling, mainly occurring* via* Leydig cells, also causes spermatogenesis arrest [35, 36]. The detection of GnRH-like material in the fluid surrounding Leydig cells that express* Gnrhr1* and the finding of* Gnrh1* mRNA in Sertoli cells [27] suggest that GnRH1 may be produced by Sertoli cells and may act as a paracrine factor to modulate the activity of Leydig cells. Here, by* in situ* hybridization, we demonstrate that in adult testis* Gnrh1* is expressed in Sertoli cells, in Leydig cells, and in peritubular myoid cells, confirming previous data concerning the activity of GnRH in the control of steroidogenesis and germ cells development as well as its release into the lumen of seminiferous tubules [2, 5]. Consistently,* Gnrh1* mRNA has also been detected in the interstitial compartment of frog testis [15] and GnRH1 positive immunostaining has recently been reported in the interstitial compartment of mouse testis during different stages of aging, with high levels observed during reproductively active stages [3]. Interestingly, the expected expression of* Gnrh1* in Sertoli cells is not widespread all over the tubules but is restricted to Stages V–VII of the spermatogenic cycle, with Sertoli cells connected to the spermatids in the last step of the maturation phase.

Present data confirm the expression of both* Gnrh1* and* Gnrhr1* in germ cells [27, 29], since the corresponding transcripts have been specifically localized in haploid cells but not in mitotic cells or in meiotic cells. Consistently, in mouse GnRH1 is an autocrine factor for gonocyte that expresses both ligand and receptors [3], but in the adult testis mitotic cells are devoid of any GnRH1/GnRHR1 immunoreactivity [3]. By contrast, in amphibians, which exhibit an annual reproductive cycle characterized by periods of spermatogenic arrest and periods of spermatogenesis onset and resumption,* gnrh1* expression has been observed in quiescent and proliferating spermatogonia as well [15].

At the end of meiosis, haploid round spermatids undergo dynamic morphologic changes, which include acrosome formation, nuclear shaping, elongation, and tail formation in order to produce sperm cells. Hence, the functional meaning of mRNA detection in haploid cells might open several interpretations. Some mRNAs repressed in early spermatids might be recruited toward the polysomes for translation in late spermatids, whereas some other mRNAs might be transcribed in a specific time window to be immediately translated. By contrast, untranslated mRNA might be degraded at specific steps during spermiogenesis or stored in sperm in order to contribute to fertilization and embryo development, with the chromatoid body thought to be the main actor in the capture of mRNAs for degradation or storage [37]. Here we demonstrate that in rat* Gnrh1* is only expressed in spermatids during the acrosome phase (Stages IX–XII), whereas* Gnrhr1* is expressed in spermatids from the acrosome phase (Stages XII-XIII) throughout the maturation phase. This is consistent with the recent finding that GnRHR1 is localized in elongating spermatids in reproductively active mouse [3]. However, the expression of ligand and receptor in the same cell type during a specific time window supports the hypothesis that GnRH1 may be an autocrine modulator of acrosome biogenesis. By contrast, the detection of* Gnrh1* mRNA in peritubular myoid cells and in Sertoli cells during the last step of the maturation phase (Stages V–VIII) in parallel to* Gnrhr1* detection in late spermatids suggests that GnRH1 may be a paracrine modulator in sperm release and transport. Consistently, in a nonmammalian vertebrate, the anuran amphibian* Pelophylax esculentus*, a species that expresses two GnRH molecular forms (GnRH1 and GnRH2) and three GnRHRs (GnRHR1, R2, and R3) [38] with a functional portioning in testis [15],* gnrhr2* mRNA has been localized in Sertoli cells, connected to elongating spermatids which express* gnrh2* [15]. In this experimental model, buserelin, a GnRH agonist, induces spermiation [39].

GNRHR1 signaling is important for sperm binding to the human zona pellucida [40] and for the inhibition of* in vivo* and* in vitro* fertilization in rodents exerted by GnRH antagonists [41]. Furthermore, GnRH-like substances have been detected in human seminal fluid and related to the acquisition of sperm functions [42], the prostate being the major candidate for its production [43, 44].

## 5. Conclusions

In rat,* Gnrh1* and* Gnrhr1* are expressed in the testis in somatic cells and in haploid germ cells. The expression of* Gnrh1*/*Gnrhr1* at specific steps of spermiogenesis and in Sertoli cells connected to spermatids in the late phase of maturation suggests a deep involvement in functions related to the production of high quality sperm.
